# Comparison of two methods for assessing diabetes risk in a pharmacy setting in Australia

**DOI:** 10.1186/1471-2458-14-1227

**Published:** 2014-11-27

**Authors:** Monique F Kilkenny, Roslyn Johnson, Nadine E Andrew, Tara Purvis, Alison Hicks, Stephen Colagiuri, Dominique A Cadilhac

**Affiliations:** Stroke and Ageing Research School of Clinical Sciences at Monash Health, Monash University, Level 1/43-51 Kanooka Grove, Clayton, 3168 Melbourne, VIC Australia; Stroke Division, Public Health, the Florey Institute of Neurosciences and Mental Health, Heidelberg, 3081 Vic Australia; The University of Melbourne, Melbourne, 3010 Vic Australia; National Stroke Foundation, Melbourne, 3000 Vic Australia; Boden Institute of Obesity, Nutrition, Exercise & Eating Disorders, The University of Sydney, Sydney, 2006 NSW Australia

**Keywords:** Hypertension, Diabetes, Cerebrovascular disease, Prevention, Health care, Risk factors, Health promotion, AUSDRISK, Random blood glucose testing

## Abstract

**Background:**

Since 2007, the Australian Know your numbers (KYN) program has been used in community settings to raise awareness about blood pressure and stroke. In 2011, the program was modified to include assessment for type 2 diabetes risk. However, it is unclear which approach for assessing diabetes risk in pharmacies is best. We compared two methods: random (non-fasting) blood glucose testing (RBGT); and the Australian type 2 diabetes risk assessment tool (AUSDRISK); according to 1) identification of ‘high risk’ participants including head-to-head sensitivity and specificity; 2) number of referrals to doctors; and 3) feasibility of implementation.

**Methods:**

117 Queensland pharmacies voluntarily participated and were randomly allocated to RBGT and AUSDRISK or AUSDRISK only. Although discouraged, pharmacies were able to change allocated group prior to commencement. AUSDRISK is a validated self-administered questionnaire used to calculate a score that determines the 5-year risk of developing type 2 diabetes. AUSDRISK (score 12+) or RBGT (≥5.6 mmol/I) indicates a high potential risk of diabetes. Median linear regression was used to compare the two measures. Staff from 68 pharmacies also participated in a semi-structured interview during a site visit to provide feedback.

**Results:**

Data were submitted for 5,483 KYN participants (60% female, 66% aged >55 years, 10% history of diabetes). Approximately half of the participants without existing diabetes were identified as ‘high risk’ based on either RBGT or AUSDRISK score. Among participants who undertook both measures, 32% recorded a high RBGT and high AUSDRISK. There was a significant association between RBGT and AUSDRISK scores. For every one point increase in AUSDRISK score there was a half point increase in RBGT levels (coefficient 0.55, 95% CI: 0.28, 0.83). Pharmacy staff reported that AUSDRISK was a simple, low cost and efficient method of assessing diabetes risk compared with RBGT, e.g. since management of sharps is not an issue.

**Conclusions:**

In a large, community-based sample of Australians about half of the participants without diabetes were at ‘high risk ‘of developing diabetes based on either AUSDRISK or RBGT results. AUSDRISK was considered to be an acceptable method for assessing the risk of diabetes using opportunistic health checks in community pharmacies.

**Electronic supplementary material:**

The online version of this article (doi:10.1186/1471-2458-14-1227) contains supplementary material, which is available to authorized users.

## Background

Diabetes mellitus (diabetes) and particularly type 2 diabetes is considered a global epidemic [1]. In Australia, the number of people diagnosed with diabetes has more than doubled during the past two decades from 1.5% (1989-90) to 4.1% (2007-08) [2]. Diabetes is an independent risk factor for cardiovascular disease (CVD). The main types of CVD are coronary heart disease, heart failure, cardiomyopathy and stroke [3]. About 11.7 million (95%) adult Australians have at least one of the major modifiable risk factors for stroke [4] including high blood pressure (BP), diabetes, high cholesterol, smoking, alcohol, poor diet, heart disease, obesity, atrial fibrillation, sleep disorders, carotid stenosis and inadequate physical activity [5]. Furthermore, knowledge of CVD risk factors is poor amongst survivors of stroke who continue to be at high risk of further events [6].

Recently, there has been a focus on increasing diabetes awareness since diabetes can be easily prevented by medication or lifestyle changes. Health promotion programs such as the Finnish Diabetes Prevention Program [7], United States Diabetes Prevention Program [8], the Australian Greater Green Triangle Diabetes Prevention Program [9] and Life! program [10] have been established to identify people at risk of diabetes. Early identification of diabetes and risk factors for diabetes means that early management and prevention programs can be provided, to reduce the potential for serious complications [11–15].

Diabetes is commonly diagnosed by either a fasting blood test or a non-fasting random blood glucose test (RBGT) taken anytime during the day. The fasting blood test is more accurate, but more time consuming and costly, and cannot be performed opportunistically. In contrast the RBGT performed by doing a finger prick test provides an immediate result and can be used to indicate increased risk of diabetes ( http://www.nlm.nih.gov/medlineplus/ency/article/003482.htm). In a study conducted by Krass et al. [16] it was found that implementation of a pharmacy screening service based on an initial risk assessment followed by a finger prick test was more cost effective in terms of numbers diagnosed with diabetes than a risk assessment only. The cost difference was driven by lower referral rates and the higher uptake of referrals following the finger prick test. However, the tick test used in this study for diabetes risk assessment was not a comprehensive diabetes risk assessment. This test, which predated the availability of current risk assessment tools, did not assess lifestyle factors such as smoking, diet or activity levels and there were no clinical measures such as waist circumference.

Diabetes risk calculation tools [17] have also been developed in the Netherlands, Thailand, Denmark, Germany, America and Finland. The most commonly used tool for identification of individuals eligible for diabetes prevention programs appears to be the FINDRISC [9]. In 2008, the International Diabetes Institute (Baker IDI) as part of the Council of Australian Governments initiative to reduce the risk of diabetes, developed and validated the Australian Type 2 Diabetes Risk Assessment Tool (AUSDRISK). This is a self-administered screening questionnaire [18] (Table 1) which calculates a score that can determine a broad level of risk of individuals developing diabetes within the next five years. The score takes into account age, gender, ethnicity, family and BP history, smoking status, diet, level of physical activity and waist measurement [18].

**Table 1:** **AUSDRISK: risk of developing type 2 diabetes within 5 years**

Risk category	Score*	5-year risk of developing type 2 diabetes*	2011 KYN diabetes (N = 1,969) n (%)
**Low**	≤ 5	1 person in every 100	251 (13)
**Intermediate**			
Intermediate	6 – 8	1 person in every 50	311 (16)
Intermediate	9 – 11	1 person in every 30	416 (21)
**High**			
High	12 –15	1 person in every 14	526 (27)
High	16 - 19	1 person in every 7	280 (14)
High	20+	1 person in every 3	185 (9)

*Source: adapted from: http://www.health.gov.au/internet/main/publishing.nsf/Content/chronic-diab-prev-aus.

Since 2007, the National Stroke Foundation (NSF) has undertaken a community-based awareness program for BP and other stroke risk factors [19, 20] called ‘Know your numbers (KYN)’. In 2011, the NSF piloted an enhanced KYN program (Pilot KYN Diabetes Program) which essentially included the use of AUSDRISK and RBGT. This program provides an opportunity for participants to identify risk factors for stroke and diabetes that are potentially modifiable and associated with health related behaviours (e.g. physical inactivity) or biomedical factors (e.g. high BP) [21]. However, it is unclear how best to measure risk of diabetes in a community based setting. In this pilot study, we aimed to compare two methods for assessing diabetes risk in pharmacy settings in Australia: random (non-fasting) blood glucose testing; and the Australian Type 2 diabetes risk assessment tool; according to 1) identification of ‘high risk’ participants including head-to-head sensitivity and specificity; 2) number of referrals to doctors; and 3) feasibility of implementation in pharmacy.

## Methods

### Pilot KYN diabetes program

**Figure 1:**
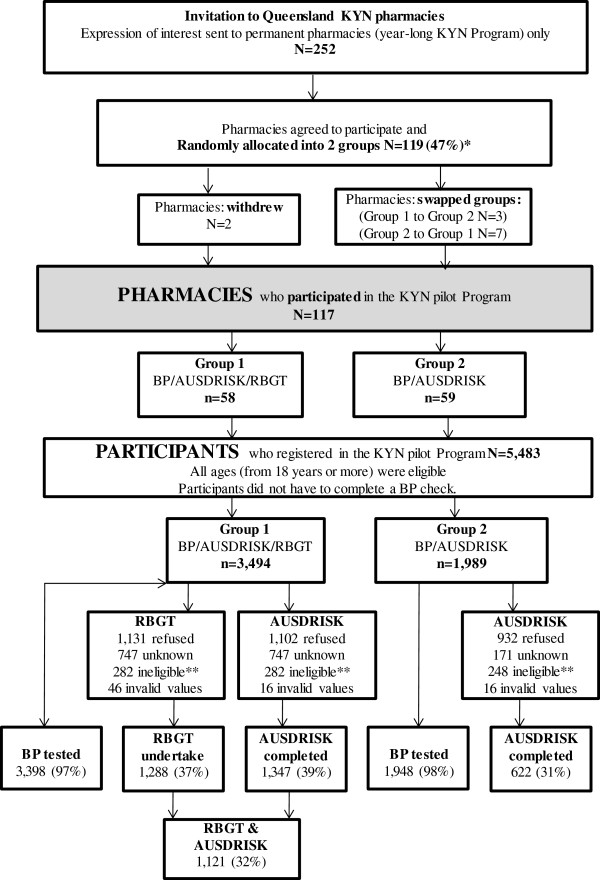
**Recruitment of pharmacies and participants who registered in KYN diabetes program.** *Pharmacies were given 2-3 weeks to change groups if they considered their allocation was not suitable **Ineligible includes people who reported having a history of diabetes. Permanent means pharmacy with year-long KYN Program; KYN: Know your numbers Program; RBGT: Random blood glucose testing; AUSDRISK: Australian Type 2 Diabetes Risk Assessment Tool.

The Queensland KYN program delivered in community pharmacies was modified in 2011 to include a free *diabetes risk assessment* and educational information for participants. A sample of KYN pharmacies in Queensland agreed to participate in the pilot study (Figure 1). These pharmacies offered the KYN program all year round on a permanent basis. The pharmacies were initially randomly allocated to provide one or two diabetes risk assessment measures in addition to a BP measurement (Figure 1). However, this was a pragmatic and voluntary study and pharmacies, although discouraged from doing so, were able to change their allocated group prior to commencement. Therefore, the study was observational and aimed to compare the two methods proposed for assessing diabetes risk as part of KYN in a pharmacy setting in Australia. The Group 1 pharmacies provided BP testing, RBGT and AUSDRISK. The Group 2 pharmacies provided BP testing and AUSDRISK only.

All staff involved in delivering the program underwent compulsory online training. The purpose of the training was to equip staff with appropriate background knowledge on stroke, diabetes and the risk factors for diabetes for CVD and provide them with clear guidelines on how to complete each of the components of the health check appropriately and safely. The training module took approximately 30 minutes to complete for pharmacies in Group 2 and 40 minutes for Group 1. An extra 10 minutes were required for Group 1 in order to provide information on risk management and infection control practices in relation to RBGT. Evaluation of the training by the participants indicated that the online training was well structured and comprehensive with sufficient and appropriate content to meet the needs of the staff providing the health checks. With respect to the format, all participants who received training commented that the program was easy and straight forward to follow. Continuous Professional Development (CPD) points were provided for those who completed the training.

### Identification of ‘high risk’ participants requiring further review

Any person who registered was included in the KYN Pilot Program. There were no eligible age range restrictions (with the exception of being 18 years or more) and participants did not have to complete a BP check. The KYN Program is targeted to people 45 years or more with training materials but this was not enforced (Figure 1). For the purposes of this paper, the term ‘high risk’ was used to classify registrants who should have been advised by pharmacists, as part of the KYN program, to seek further review and assessment of their risk of developing diabetes or CVD (i.e. if BP ≥140/90 mm Hg). The definitions for high risk of diabetes or BP was in keeping with Australian clinical guideline recommendations [22] (e.g. Royal Australian College of General Practitioners guideline for diabetes management in general practice endorsed by Diabetes Australia http://www.racgp.org.au/your-practice/guidelines/diabetes/) available at the time this study was undertaken. Participants were classified as ‘high risk’ of developing diabetes within the next 5 years if they had an AUSDRISK score (12+) or RBGT (≥5.6 mmol/I). These risk classifications were agreed by the KYN Advisory Committee which had representation from Diabetes Australia, Pharmacy Guild of Australia, NSF, pharmacists, clinical experts and researchers. The participants who were identified as being ‘high risk’ were provided with a standard letter for their local doctor which included their risk assessment results undertaken as part of the KYN program.

### Sensitivity and specificity of AUSDRISK and RBGT

In order to measure the discrimination potential of AUSDRISK and RBGT we mapped the consistency of AUSDRISK and RBGT scores for people who undertook both measures using the AUSDRISK as the "reference standard". Based on the available data and its distribution features median regression with bootstrapping adjusting for age and gender was used to compare the RBGT levels with AUSDRISK scores. Further analyses included calculating the sensitivity, specificity, positive and negative predictive value for all participants and by specific age groups (<55 years and 55 years or more).

### Quantitative diabetes risk data collection and analyses

All pharmacies collected data on a registration log. People who registered to have their risk of diabetes and BP measured as part of the Pilot KYN Diabetes Program were asked to provide basic demographic information, such as age, gender, history of diabetes and or high BP. The registration log was also used to record the results of risk assessments (AUSDRISK score and or RBGT level), BP readings (systolic and diastolic blood pressure), and whether a referral to a general practitioner had been recommended for ‘high risk’ participants by the pharmacy staff.

Chi-square test for categorical variables was used to compare groups for age, gender, ‘high risk’ categories and number of referrals. Shapiro-Wilk test was performed to test data for normality. Median and quartiles (25^th^ and 75^th^) values were calculated for systolic and diastolic BP readings, AUSDRISK and blood glucose scores. Kruskal-Wallis test was used to compare Group 1 and Group 2. Level of significance was set up at p < 0.05. All quantitative analyses were performed using Intercool STATA 12.1 for Windows software (Stata Corp PL, 2013).

### Feasibility of implementation in pharmacy

As part of the evaluation for the pilot a NSF project officer (AH) collected qualitative data during site visits to pharmacies (Additional file 1). A detailed semi-structured interview scheduled was developed (AH, DC, MK, RJ). The interview schedule included both open and closed questions relating to feedback on the pilot program. For example, there were questions to explore the impact on staff time and resources, space to conduct the KYN Program, safety considerations, use of consumables, feedback on procedures and sustainability of undertaking the different diabetes risk assessments, where applicable.

All participating pharmacies were contacted to complete the interview. To be eligible for an interview, a pharmacy needed to have commenced delivering the program. The primary contact in the pharmacy responsible for implementation of the pilot program ("the champion" who was usually the pharmacist or pharmacy assistant) was requested to participate in the interview. The number of questions asked depended on time availability and the local context. The same researcher (AH) conducted all the interviews. The data were directly recorded on a paper-based interview schedule by the interviewer (AH). After which, de-identified data were then entered into SurveyMonkey ( http://www.surveymonkey.com) for ease and accuracy of recording.

The qualitative free text data were then transferred to Excel (Microsoft Office Corp, 2010) and subjected to thematic analysis. Using an inductive approach for the analysis, a coding tree outlining the major themes was developed and used to systematically code and then analyse the text responses. Responses were independently coded by two researchers (AH and TP) and then the results were cross-checked. Any discrepancies noted in the coding were discussed and resolved. Once no new information was being elicited from the completed interviews they were ceased since saturation of information had been reached [23].

Ethics approvals for the evaluation were provided by the Austin Health Human Research Ethics Committee (H2007/03028) and Queensland Health Central Office Human Research Ethics Committee (HREC/11/QHC/8).

## Results

### Participation of pharmacies

Overall 117 pharmacies were randomly allocated to provide one or two diabetes risk assessment measures in addition to a BP measurement (Figure 1 and Table 2) as part of conducting their KYN program between May and September in 2011. The participation rate and number of participants per site was greater in Group 1 than Group 2 pharmacies (Figure 1). Nearly all participants at pilot pharmacies had a BP measurement recorded, while one in three participants had a diabetes risk assessment recorded.

**Table 2:** **Comparison of people tested in the KYN diabetes program by group 1 (BP/AUSDRISK/RBGT) and group 2 (BP/AUSDRISK)**

	Group 1 BP/AUSDRISK/RBGT n (%)	Group 2 BP/AUSDRISK n (%)	Total n (%)
Number of registered health check stations	58	59	117
Number of registration logs returned	3,494	1,989	5, 483
Demographics			
Female	2,023 (59)	1,203 (62)	3,266 (60)
Age 55 years or more	2,233 (66)	1,305 (67)	3,538 (66)
Do you have diabetes?			
Yes	282 (11)	248 (16)*	530 (13)
No	1,956 (76)	1,164 (74)	3,120 (75)
Not sure	334 (13)	164 (10)	498 (12)
AUSDRISK score			
Median (Q1, Q3)	12 (8, 15)	12 (8, 15)	12 (8, 15)
Low (<6)	158 (12)	93 (15)	251 (13)
Intermediate (6-11)	514 (38)	213 (34)	727 (37)
High (12+)	675 (50)	316 (51)	991 (50)
*12-15*	*351 (26)*	*175 (28)*	*526 (27)*
*16-19*	*193 (14)*	*87 (14)*	*280 (14)*
*20+*	*131 (10)*	*54 (9)*	*185 (9)*
RBGT			
Median (Q1, Q3)	5.8 (5.0, 6.9)	-	5.8 (5.0, 6.9)
Normal (less than 5.6 mmol/l)	602 (47)	-	602 (47)
High (5.6 mmol/l or more)	686 (53)	-	686 (53)
‘High risk’ of diabetes^1^	818 (74)*	316 (51)	1,134 (66)
BP readings			
Systolic median (Q1, Q3)	135 (123,149)	136 (123,150)	135 (123,150)
Diastolic median (Q1, Q3)	81 (73, 89)	81 (73, 90)	81 (73, 90)
BP categories#			
Normal	553 (16)	290 (15)	843 (16)
High-normal	1,272 (37)	707 (36)	1,979 (37)
High	1,573 (46)	951 (49)	2,524 (47)
Knowledge of BP number			
Reported no history of high BP	1,065 (38)*	540 (33)	1,605 (36)
Referral to GP	1,441 (49)*	682 (37)	2,123 (44)
Recorded High BP check			
Reported no history of high BP	337 (25)**	174 (21)	511 (23)
Referral to GP			
Recorded ‘High risk’ of diabetes^1^	501/776 (65)	220/297 (74)*	721/1,073 (67)
Recorded High BP	900/1,328 (68)*	482/886 (54)	1,382/2,214 (66)

BP: blood pressure; AUSDRISK: Australian Type 2 Diabetes Risk Assessment Tool; Q1: 25^th^ percentile; Q3: 75^th^ percentile; High normal: 120-139/80-89 mm Hg; High: ≥140/90 mm Hg; ‘1; ‘High risk’ of diabetes (AUSDRISK score 12+ or RBGT ≥5.6 mmol/I); *p < 0.05 and **p < 0.07; #isolated systolic BP not reported since this represents <0.3% of data.

### Profile of participants

Data were returned for 5,483 KYN health checks (Figure 1). More females participated in the Pilot KYN Diabetes Program compared to males (Table 2). This level of participation by gender was consistent across the various age groups, and about two-thirds of the participants were aged 55 years or more. There were no age or gender differences between participants attending Group 1 and 2 pharmacies. Over half (58%) of participants were opportunistically ‘passing by’ the pharmacy which motivated their decision to have a KYN health check.

Just under half (47%) of participants recorded a high BP (≥140/90 mm Hg) (Table 2). Of those participants with a high BP reading recorded, 23% reported no history of high BP and 66% were referred to their general practitioner for further assessment (Table 2). One in ten participants reported a history of diabetes. Participants attending Group 2 pharmacies were more likely to report a history of diabetes (16%) than participants attending Group 1 pharmacies (11%, p < 0.05).

Eligible participants (excluding those with a history of diabetes) attending Group 1 pharmacies were more likely to complete AUSDRISK (39%) than participants attending Group 2 pharmacies (31%, p < 0.05). BP testing was undertaken by nearly all participants in Group 1 (97%) and Group 2 (98%) pharmacies.

### Identification of ‘high risk’ participants

**Figure 2:**
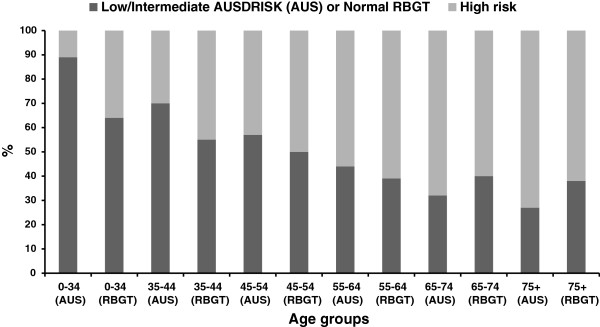
**High risk scores (AUSDRISK and RBGT) by age groups.**

Overall 1,969 participants without a reported history of diabetes had an AUSDRISK assessment undertaken. Table 2 shows that 50% of these participants had high AUSDRISK scores. Figure 2 shows the proportion of people by age group within various AUSDRISK categories. Males were more likely to have a high AUSDRISK score than females (AUSDRISK 12+: males 58%, females 46%, p < 0.001). There were no differences between participants attending Group 1 and 2 according to AUSDRISK scores.

Overall 1,288 participants without a reported history of diabetes had a RBGT. Fifty-three per cent of these participants had a high RBGT level. There was no difference between RBGT level according to gender (RBGT level ≥5.6 mmol/l: males 54%, females 53%, p = 0.55). Figure 2 shows the proportion of people by age group within ‘high risk’ RBGT categories.

### Referral of participants with ‘high risk’ of diabetes to general practitioners

As part of the KYN Program, pharmacists were educated that all participants determined to be at ‘high risk’ of diabetes should be referred to their general practitioner for further assessment. However, approximately one-third of participants identified as having a ‘high risk’ of diabetes were not referred to their general practitioner. However, we do not know if participants were not referred because they were already seeing a doctor for ongoing monitoring of these risk factors. Participants attending Group 1 pharmacies were less likely to be recommended to see their general practitioner for further assessment if they were at ‘high risk’ of diabetes compared to participants attending Group 2 pharmacies (Group 1 65%, Group 2 74%, p = 0.003).

### Sensitivity and specificity

Overall, 1,121 participants without a history of diabetes had both diabetes risk assessment measures (RBGT and AUSDRISK). About one third of participants (369/1,121) were identified at high risk of diabetes (by both AUSDRISK and RBGT). Of the 589 participants with high AUSDRISK score, 369 (63%) also had a high RBGT level and 220 (37%) had a normal RBGT level. Of the 532 participants with low to intermediate AUSDRISK score, 244 (46%) also had a high RBGT level and 288 (54%) had a normal RBGT level.

**Figure 3:**
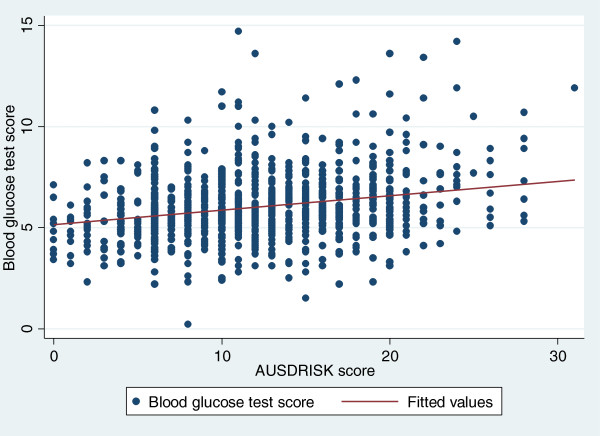
**Relationship of AUSDRISK and random blood glucose test measures using median linear regression.**

Of the participants who undertook both measures, 33% had both high RBGT and high AUSDRISK; 20% normal RBGT and high AUSDRISK; 22% high RBGT and low AUSDRISK; 26% normal RBGT and low AUSDRISK. Using median linear regression with bootstrapping (Figure 3) adjusting for age and gender we showed that for every one point increase in AUSDRISK score there was a half point increase in RBGT levels (coefficient 0.55, 95% CI; 0.28, 0.83). Using AUSDRISK as the reference standard the sensitivity and specificity of the RBGT test was low (Table 3). The AUSDRISK score was less specific for age group (55+ years).

**Table 3:** **Sensitivity, specificity and predictive values of RBGT (**≥**5.6 mm/mol) to predict risk of diabetes at various age categories (AUSDRISK 12+ is the reference standard)**

Variables	All ages N = 1,121 % (95% CI)	Age <55 years N = 453 % (95% CI)	Age 55+ years N = 661 % (95% CI)
Sensitivity	63 (59, 67)	63 (54, 71)	63 (58, 67)
Specificity	54 (50, 58)	62 (56, 68)	43 (37, 50)
Positive predictive value	60 (56, 64)	43 (36, 50)	69 (64, 74)
Negative predictive value	57 (52, 61)	79 (73, 83)	36 (30, 42)

### Feasibility of implementation in pharmacy

Qualitative feedback was obtained from 68 pharmacy staff: 38 in Group 1 and 30 in Group 2. Respondents were either a pharmacist or pharmacist assistant. The major themes that emerged from the interviews related to time requirements, staff competency, the procedure itself, and referrals to general practitioners (Table 4). Aspects such as limited staff and consumer time and staff training reportedly affected the number of participants who received a diabetes risk assessment in both Groups:

*"Time impacts our ability to complete the entire health check…"*

*"Customers are usually very rushed……therefore* [they are] *more likely to just want to have a BP check….."*

**Table 4:** **Main themes and sub-themes to emerge from interviews with pharmacy staff related to conducting diabetes risk assessments**

Theme and sub-themes		Group 1* (N = 38)	Group 2** (N = 30)
**Time constraints**			
Training	Only selected staff were allocated time to complete the training. Therefore, if they haven’t completed the training, they may just completed BP check	✓	✓
Training/ access to equipment	By the scheduled start date for the pilot phase, some pharmacies were still waiting on a RGBT meter and training regarding its use	✓	
Length of assessment (pharmacy staff)	Total health check more likely to take >5 or <5 minutes	>5 minutes	<5 minutes
	Therefore, an appointment often had to be made to complete (>5 minutes)		
Pharmacy staff	If staff had limited time, often only a BP check would be completed	✓	✓
Length of assessment (participants)	Limited available time of participants affected the number of diabetes risk assessments completed	✓	✓
**Competency of staff carrying out health check**			
Pharmacy staff undertaking measures	Health checks more likely to be performed by fully trained pharmacist	✓	
	BP and AUSDRISK checks more likely to be completed by pharmacist assistants		✓
Pharmacy staff attributes	Concerns raised regarding confidence, knowledge, skill and experience of a pharmacy assistant in carrying out RBGT	✓	
	When a pharmacy assistant performed AUSDRISK, high risk patients were generally discussed with the pharmacist		✓
Motivation	Lack of motivation by staff limited how often the diabetes risk assessment was completed	✓	✓
**Procedure**			
Space	Limited number of pharmacies had a designated ‘booth’ or private consulting room to complete RBGT. General feeling that it was inappropriate to conduct RBGT test in a pharmacy setting	✓	
	Set up of a pharmacy in general was more conducive to completion of the AUSDRISK assessment than a RBGT		✓
Technique/participants	Invasive nature of finger prick as part of RBGT was a concern for some participants	✓	✓
	Some consumers refused waist measurement as part of AUSDRISK		
Safety and cost	Concerns around appropriate disposal of ‘sharps’ and ‘clinical waste’ involved in RBGT	✓	
	Purchase of consumables required to undertake RBGT and for infection control measures are expensive	✓	
Referral to GP	If blood glucose level was >5.5 mmol and the consumer had recently eaten or had something sweet to drink, staff reported that the may not refer, but rather request to re-assess the participant at a later time point	✓	
	If AUSDRISK score was high due to non-modifiable risk factors (eg age and gender) staff were less likely to refer		✓

AUSDRISK: Australian type 2 diabetes risk assessment: BP: Blood pressure; RBGT: Random blood glucose testing; *BP/AUSDRISK/RBGT; ** BP/AUSDRISK.

However, it was generally perceived that the AUSDRISK was able to be performed by a pharmacy assistant unlike the RBGT in many pharmacies *"…some PAs* [pharmacy assistants] *are not confident with BGT and are not interested in learning*". Respondents also felt the AUSDRISK was safer to perform, less invasive, less time consuming, and was not as costly as performing a RBGT.

"….*you need a designated area* [for the RBGT]….*and the storage and cost of all consumables required for the blood glucose test makes it difficult*"

"*Need to take the customer away to a private consulting room* [for RBGT]*"*

## Discussion

The Pilot KYN Diabetes Program was successful in comparing the feasibility of two methods for assessing diabetes risk in a community setting. Both methods were successful in being able to identify people in the community with an increased risk of developing diabetes within the pharmacy setting. We found that about one in two participants were at risk of developing diabetes (50% of participants had high AUSDRISK score and 55% a high RBGT levels). These findings suggest important implications for a program such as KYN and demonstrate the need for improved awareness, detection and management of people at risk of type 2 diabetes. The AUSDRISK tool was more acceptable to staff than providing the combined testing and resulted in more referrals of participants with ‘high risk’ of diabetes to general practitioners. The Pilot KYN Diabetes Program provided the opportunity for over 2,000 participants to improve their awareness of diabetes as a risk factor for stroke and other CVD risk factors. The KYN Program also resulted in over 645 people being recommended to seek further consultation with their general practitioner for a comprehensive assessment of their diabetes risk and CVD risk status.

This is one of the largest studies to have used the AUSDRISK tool in a pharmacy setting in Australia. The use of the AUSDRISK is recommended as part of the Australian National Evidence Based Guideline for the Primary Prevention of Type 2 Diabetes [24]. The tool has been validated in two independent Australian cohorts (the Blue Mountains Eye Study of 2123 participants [25] and 4060 participants in the North West Adelaide Health Study) [26]. AUSDRISK has also been validated in the Geelong osteoporosis (n = 1,404) study [27]. These previous studies were designed to validate the AUSDRISK against results from a fasting blood test or fasting oral glucose tolerance test. However, Wong and colleagues found that the use of AUSDRISK in general practice was low [28]. The Melbourne Diabetes Prevention Study is using AUSDRISK for assessment of eligibility for a randomized controlled trial [10] and will be the first study to compare AUSDRISK with FINDRISC as diabetes risk assessment tools. AUSDRISK is also routinely used in the LIFE! program which has now assessed diabetes risk for 8,412 people [29]. We provide important new information on use of AUSDRISK as part of a well-established health promotion program in the community.

The Pilot KYN Diabetes Program was used to test two approaches through offering AUSDRISK or AUSDRISK/ RBGT. We undertook a ‘per program’ analysis since this was the most appropriate method for answering our research questions in this pragmatic study. We found that pharmacies offering AUSDRISK/RBGT attracted a greater number of participants (n = 3,494 vs n = 1,989) and more people who participated in diabetes risk assessments (39% vs 31%, respectively), when compared to pharmacies offering AUSDRISK only. It is unknown whether participants were aware before being tested which approach was being offered. Greater participation in the approach in Group 1 (AUSDRISK/RBGT) may be due to participant perceptions including the fact that people may more strongly associate diabetes testing with a finger prick test. A study conducted by Krass et al. [16] found that implementation of a pharmacy screening service based on an initial risk assessment followed by a finger prick test was more cost effective in terms of numbers diagnosed with diabetes than a risk assessment only. The cost difference was driven by lower referral rates and the higher uptake of referrals following the finger prick test. In our study we are unable to test this as we have no follow-up data.

Random allocation of pharmacies was not stratified and pharmacies could swap groups prior to commencing. The pharmacies electing to move into Group 1 were predominantly National Diabetes Services Scheme pharmacies seeking to learn a new skill and offer an additional service to their customers (i.e. blood glucose testing) or were currently offering blood glucose tests and were keen to continue with this service. Some selection bias was noted following review of these findings. There was tendency for the more active pharmacies in the KYN Program to be offering AUSDRISK/ RBGT (Group 1), and this may have positively influenced participation. Using data from the KYN 2010 Program, Group 1 pharmacies were more likely to have previously participated in the Pilot KYN Diabetes Program (Group 1: 67%, Group 2: 59%) and undertook greater volumes of checks (mean number of checks: Group 1: 157; Group 2: 62). Future studies would need to stratify pharmacies by previous participation in KYN and participant volumes before randomisation in order to account for these differences. Pharmacies should also be kept blinded to options so there are no crossovers.

One of the primary purposes of the KYN Program is the referral of potentially ‘high risk’ people to their general practitioner. It is also about raising awareness of stroke risk factors and diabetes. Overall, Group 1 had greater participation and higher number of referrals for participants with ‘high BP’ compared with Group 2 participants. However, the participants in Group 2 with ‘high risk’ of diabetes were more likely to be recommended to follow-up with a general practitioner compared with Group 1 participants. Specifically in relation to this study, staff of Group 2 pharmacies may have had more time to discuss their results with participants and provide referrals than those in Group 1. Another potential source of reporting bias is that the differences in number of referrals between Group 1 and Group 2 could be because the referral may have been given verbally by pharmacy staff and not documented on the registration log. The majority of staff also felt that the cut-off of the RBGT for referral was too low at >5.5 mmol.L which may have influenced referral rates. Future studies should include information on why the participant was not referred, for example, if they already visit their doctor regularly; are being managed for their current health problem; or whether the participant refused a referral letter.

As with all risk assessment tools, AUSDRISK and RBGT have limitations. The AUSDRISK has been validated in several studies [25–27] and is recommended for use in the Australian setting [24]. However, there is emerging evidence that the cut-points chosen in our pilot project of 5.6 for RBGT and 12+ for AUSDRISK may be too insensitive for efficiently identifying people who may go on to develop diabetes [10]. This means that a larger number of people may have been considered as requiring a referral to their doctor, than was actually necessary, to ensure a cost-effective health promotion program. In future reviews of the KYN program it will be important that cut-points for referral remain consistent with evidence–based national guideline recommendations or compelling new research relevant to the field. This ensures that the most appropriate diabetes risk screening approaches suitable for the setting in which they are applied are used, and that primary care resource use is optimised (i.e. inappropriate referrals to general practice are minimised).

The sensitivity and specificity of the RBGT against the AUSDRISK was low. This may be because AUSDRISK and RBGT are two different diabetes risk assessment measures. AUSDRISK measures risk of developing diabetes within the next five years (future risk). RBGT can be used to assess risk of having undiagnosed diabetes. We found that two-thirds of participants were at high risk of diabetes (defined as high AUSDRISK and or high RBGT). When we compared the participants with high AUSDRISK score two in three also had a high RBGT level. When adjusted for age and gender there was an association between the two measures. We found that for every one point increase in AUSDRISK score there was a half point increase in RBGT levels. The implications of these findings for a health promotion program are that RBGT would not be a reasonable proxy for diabetes risk assessment (such as AUSDRISK) for 37% of participants. To our knowledge there have been no other studies that have compared RBGT and AUSDRISK tools.

The additional qualitative data provided evidence to support the use of the AUSDRISK only instead of RBGT. The RBGT and AUSDRISK approach (Group 1) required more staff time and resources. Infection control procedures, additional training and the extra consumables required for RBGT make it less cost-effective for implementation by community pharmacists as part of a health promotion program. A weakness of this pilot program was that no formal cost-effectiveness evaluation or registrant feedback on the different methods of assessing diabetes risk was conducted. Consumer feedback on different methods of assessing diabetes risk was only indirectly obtained from pharmacists who reported that KYN registrants felt that RBGT was more invasive. The roles of pharmacy assistants are important in the sustainable role out of health promotion programs in pharmacies, and there were common concerns with the skill, experience and confidence of pharmacy assistants to undertake a RBGT and the impracticalities of this measure. Alternatively, AUSDRISK is a simple and efficient measure to assess people’s risk of diabetes in a community setting.

The potential limitations of the KYN data have been noted in previous publications in related to the KYN Program [19, 20]. In brief, the group of participants being tested was a convenience sample and may not be representative of the general population. In relation to this study specifically, we were only able to assess diabetes risk for around a third of the participants and for the group tested for risk of diabetes we were unable to collect outcome data on diabetes status. The pharmacist recommendation is a proxy outcome for doctor attendance and detection of undiagnosed diabetes or participation in the Life! Program. In order to fully compare the two diabetes risk assessment measure (AUSDRISK and RBGT) we need to know who actually developed diabetes. The pilot study could have been strengthened if it had been possible to follow-up with general practitioners for all ‘high-risk’ registrants that visited their doctor based on the results of their KYN determined diabetes risk status. Among KYN registrants who are referred to their doctor, 85% have self-reported visiting their doctor [19, 20]. It would also have been strengthened if the results from RBGT and AUSDRISK were compared to the gold standard (e.g. fasting blood test or oral glucose tolerance test) for a sample of participants and pharmacies (stratified according to size, experience of staff to the Program). The strengths of our study include the large sample of pharmacies and community-based participants.

## Conclusions

The Australian Pilot KYN Diabetes Program provides evidence of the feasibility and acceptability of a method of raising community awareness of type 2 diabetes in community pharmacy locations. Despite some advantages and disadvantages of providing both diabetes tests AUSDRISK alone was deemed to be the preferred method since providing two tests added to the complexity of the program for pharmacy staff. The program provided an opportunity for people to have their risk for type 2 diabetes assessed and understand their results. Participants classified at ‘high risk’ of diabetes were more likely to be referred to their doctor for a comprehensive assessment. As a result of the Pilot KYN Diabetes Program a recommendation was made by the KYN Advisory Committee to include a diabetes risk assessment using the AUSDRISK in conjunction with BP testing as a permanent feature of the KYN Program. In 2013, 40,780 KYN participants had received an AUSDRISK assessment (46% of participants with high AUSDRISK score 12+) from approximately 1500 pharmacies across two states (Queensland and New South Wales) of Australia.

## Authors’ information

MK (MPH, Senior Research Officer)

RJ (MPH, National Know your numbers Manager)

NA (PhD, Research Fellow)

TP (MSci, Research Officer)

AH (Grad Dip Hth Prom, Queensland Know your number Coordinator)

SC (MBBS FRACP, Professor of Metabolic Health)

DC (PhD, Head and Principal Investigator).

## Electronic supplementary material

Additional file 1: **Extended diabetes pilot: pharmacy site questionnaire.** (DOCX 27 KB)

## Abbreviations

AUSDRISK: Australian diabetes risk assessment tool
BP: Blood pressure
CVD: Cardiovascular disease
IDI: International Diabetes Institute
NSF: National Stroke Foundation
Q1: Quartile 1 (25^th^ percentile)
Q3: Quartile 3 (75^th^ percentile)
RBGT: Random blood glucose test (non-fasting)
SD: Standard deviation.
