# Supplementary material for: Comparison of two methods for assessing diabetes risk in a pharmacy setting in Australia
Source: BMC Public Health. 2014 Nov 27;14:1227. doi: 10.1186/1471-2458-14-1227 (PMC4289299; doi:10.1186/1471-2458-14-1227)
Supplement: Supplementary file 1 — Additional file 1: Extended diabetes pilot: pharmacy site questionnaire. (DOCX 27 KB) [file 12889_2014_7399_MOESM1_ESM.docx]

# Extended Diabetes Pilot: Pharmacy Site Questionnaire

| Pharmacy Name & ID |  |
| --- | --- |
| Address: |  |
| Phone Number: |  |
| Date of Visit / Phone Call |  |
| Staff Member contacted: |  |
| Program participating in: |  |

Is there a dedicated point of contact in the pharmacy for the KYN program? And comment on the communication process within the pharmacy.

## Online Training Module

Did all staff involved in completing the health checks complete the online training? Yes / No

How many staff completed the training?

Did the staff complete the training prior to offering the health checks? Yes / No

Were there any barriers to completing the online training in a timely manner?

What was the occupation/qualification of the staff members involved in the KYN health checks?

- Pharmacist
- Intern Pharmacist
- Pharmacist assistant
- Other

Was there any concern over the required skill level to complete the health checks?

What do you believe were the benefits /advantages of the online training module in the KYN program?

Suggestions for improvement

***RBGT – Training***

Did you receive training in use of the Optium Xceed meter prior to commencing the pilot? Yes/ No

- Face to Face
- Telephone

Do you believe the training was appropriate?

Suggestions for improvement

## Process

Is there a dedicated area or booth for conducting the health checks? Yes/ No

Who usually initiates the request for a KYN check?

- Customer
- Pharmacist / Pharmacy Assistant
- About half and half

What is the average duration of a Group 1 health check (including counselling and provision of resources)?

What is the average duration of a Group 2 health check (including counselling and provision of resources)?

How many health checks per week would your pharmacy take on average?

Are you logging the health checks on the Registration Log / IT platform?

□ Always □ Most of the time □ Occasionally □ Never

What are the barriers to returning the completed registration logs?

What were some of the reasons for refusal to complete the AUSDRISK?

What were some of the reasons for refusal to complete the RBGT?

***Consumer Resources:***

Are the following resources discussed with the customer?

|  | Always | Most of the time | Occasionally | Never |
| --- | --- | --- | --- | --- |
| Brochure |  |  |  |  |
| 123 Action Plan |  |  |  |  |
| GP Referral Letter |  |  |  |  |

If the resources are not being used, why?

How easy was the Action Plan to use during the health check?

Do you refer a customer to their Doctor if they were identified as being “high risk”

□ Always □ Most of the time □ Occasionally □ Never

If not “Always”, why are customers not referred?

Have Doctors ever given you feedback about the KYN program via your customers once they have been referred on? Or identified at high risk?

## Point of Sale Resources

Are the following promotion resources used within the store?

|  | Yes | No |
| --- | --- | --- |
| A4 counter display |  |  |
| A2 poster |  |  |
| Shelf Wobbler |  |  |
| Prescription reminder card |  |  |
| Hanging Ceiling poster |  |  |
| Pharmacy Assistant Badge |  |  |
| Window Banner |  |  |
| Window Sticker |  |  |

If resources are not being used, why?

What if any additional promotional materials would you like to see or work well within a pharmacy?

Is the pharmacy currently offering any other health checks? Yes/ NO

## IT platform

Did you use the electronic survey tool during the pilot? Yes/ No

If “no” why? Was alternative software used?

Do you have a designated computer for the health checks? Yes/ No

Did you find using the tool made the health checks more efficient?

Was the initial support and training in the use of the software adequate?

Any suggestions for improvement?

## Customer feedback

What feedback do you receive from customers the pilot program?

## Pharmacy benefits of project involvement

What do you see are the benefits for your pharmacy of being involved with KYN?

What do you identify the barriers to participation in the Enhanced Pilot?

Any suggestions on how the program could be improved?

Any suggestions for improvement of support offered by the NSF?
